# Application of ICEEMDAN Energy Entropy and AFSA-SVM for Fault Diagnosis of Hoist Sheave Bearing

**DOI:** 10.3390/e22121347

**Published:** 2020-11-28

**Authors:** Ziming Kou, Fen Yang, Juan Wu, Tengyu Li

**Affiliations:** 1 College of Mechanical and Vehicle Engineering, Taiyuan University of Technology, Taiyuan 030024, China; wujuan@tyut.edu.cn (J.W.); litengyu0001@link.tyut.edu.cn (T.L.); 2 School of Mechanical Engineering, North University of China, Taiyuan 030051, China; nucmyf125@nuc.edu.cn

**Keywords:** sheave bearing, energy entropy, support vector machine, artificial fish swarm algorithm

## Abstract

The mine hoist sheave bearing is a large heavy-duty bearing, located in a derrick of tens of meters. Aiming at the difficulty of sheave bearing fault diagnosis, a combined fault-diagnosis method based on the improved complete ensemble EMD (ICEEMDAN) energy entropy and support vector machine (SVM) optimized by artificial fish swarm algorithm (AFSA) was proposed. Different location of the bearing defect will result in different frequency components and different amplitude energy of the frequency. According to this feature, the position of the bearing defect can be determined by calculating the ICEEMDAN energy entropy of different vibration signals. In view of the difficulty in selecting the penalty factor and radial basis kernel parameter in the SVM model, the AFSA was used to optimize them. The experimental results show that the accuracy rate of the optimized fault-diagnosis model is improved by 10% and the diagnostic accuracy rate is 97.5%.

## 1. Introduction

The head sheave is the key part in the multi-rope friction winder. The bearings of the head sheave are subjected to the heavy and complex loads. In case of failure, it will bring huge security risks and economic losses to the coal mine. Therefore, it is pressing to study the fault-diagnosis methods of head sheave bearings. Since the sheave bearings of mine hoist are characterized by low speed (40 rpm to 60 rpm) and heavy load (e.g., tens or hundreds of kN), the impacts caused by the sheave bearing faults are weak. So, it is difficult to achieve ideal results with traditional fault-diagnosis methods. At present, the fault diagnosis of head sheave bearings mainly relies on workers to climb to the dozens of meters high platform, which is very dangerous, especially in extreme weather. Besides, monitoring the temperature of bearings is also very common in coal mine to get fault information, which indicates the bearing may have faults according to the excessive high temperature, but it cannot predict the exact position of the fault. The vibration-based fault diagnosis can avoid the shortcomings of monitoring temperature, and have been researched by many scholars by signal processing [1,2,3,4]. In literature [5], a fault features extraction scheme based on MED-ICEEMDAN, mutual information, and sample entropy was proposed for the head sheave bearing vibration signals. Huang et al. [6] used the morphological filtering method to analyze the vibration signals whose operators and structure elements are optimized by the particle swarm algorithm to diagnose the faults of railway vehicle bearing. The adaptive multipoint optimal minimum entropy deconvolution adjusted method was proposed in the paper [7] to extract fault-related features from noisy vibration signals. In the literature [8], the authors used the sparsity within and across groups property of the bearing fault signals in frequency domain, combined with the moth-flame optimization, to extract the fault feature that can identify the condition of the bearing. A fault feature extraction method based on the improved compound interpolation envelope local mean decomposition and fast kurtogram was introduced for wheelset bearings in the paper [9]. The literatures [10,11] made use of the acoustic signals of commutator motors with different faults that can be detected by signal-processing methods.

The fault-diagnosis method based on signal processing to extract fault characteristic information is defective as it needs to be executed and completed by professionals with professional knowledge and technology, and cannot provide the diagnosis results in time. It may not only delay the time to repair the fault, but may even cause serious accidents and casualties. Therefore, providing an intelligent fault-diagnosis method is of great significance for ensuring the safety of coal mine production. As one of the most intelligent and cutting-edge fields in the field of artificial intelligence, the application of SVM received increasing attention [12,13,14], reflecting in the aspects of regression estimation, pattern recognition, and fault diagnosis, such as the fault diagnosis of the vehicle suspensions, automatic detection of diabetic eye disease, and predictive control of the industrial process [15,16,17]. In the aspect of bearing fault diagnosis, the application of SVM has been reported in many literatures [18,19,20,21]. For example, Gu et al. [18] proposes an approach based on the variational mode decomposition, support vector machine, and statistical characteristics to analyze the vibration signals of bearing on the spindle device of the mine hoist. Li et al. [19] used ensemble SVM for the intelligent classification of the bearing’s faults, combined with the nonlinear dynamics entropy. Van et al. [20] proposed a hybrid fault-diagnosis method for bearing based on the particle swarm optimization and least squares wavelet support vector machine, whose feature vectors are obtained by minimum-redundancy maximum-relevance method. In the paper [21], the authors used the time and frequency domain features as the feature vectors of the support vector machine for early detection and classification of bearing faults in electrical motors and generators. To improve the classification accuracy of multiclass support vector machines, the authors in paper [22] introduced a dynamic reliability measure technique for individual support vector machines, and achieved good results for the experimental data. In literature [23], three faults (headframe inclination, bearing faults, and head sheave swing) of the head sheaves are investigated by boosted tree, K-nearest neighbor, Naive Bayes classification and support vector machine, and five neural networks, respectively, which show that the support vector machine without optimization has poor performance.

From the above literatures, it can be seen that the fault-diagnosis research on sheave bearings are relatively few and lack the intelligent fault-diagnosis research on sheave bearings. Our aim is providing an intelligent fault-diagnosis method with high accuracy. The main factors affecting the accuracy of the diagnostic system are the extraction of feature information and the setting of model parameters. This paper used the energy entropy of the intrinsic mode functions (IMFs) containing the main fault information to establish feature vector, according to the fact that different bearing faults have different ICEEMDAN energy entropy, and uses the artificial fish swarm algorithm to optimize the penalty factor and radial basis kernel parameter in the support vector machine model to obtain the optimal diagnostic model. The experimental results show that the accuracy rate of the optimized fault-diagnosis model is improved by 10% and the diagnostic accuracy rate reached 97.5%.

## 2. Feature Extraction of Bearing Vibration Signals Based on ICEEMDAN Energy Entropy

During the establishment of the diagnostic model, the feature information is related to the accuracy and reliability of the diagnosis results, and the extracted feature information must be able to reflect the nature of the corresponding state. The improved complete ensemble empirical mode decomposition with adaptive noise (ICEEMDAN), as an improvement on empirical mode decomposition (EMD), is an adaptive processing method for signals. Its essence is to decompose a complex signal into a finite number of intrinsic mode functions with transient frequencies. These IMFs contain the detailed characteristics of the signal and can essentially reflect bearing’s corresponding state.

For rolling bearings, the faulty part may be the bearing inner race, outer race, ball, and cage. The different faulty parts will generate different vibration signals with different frequencies, which will result in different IMFs after ICEEMDAN decomposition. Therefore, it is an ideal choice to use the IMFs obtained by ICEEMDAN decomposition of the signal to construct feature information.

### 2.1. Improved Complete Ensemble EMD

**Figure 1:**
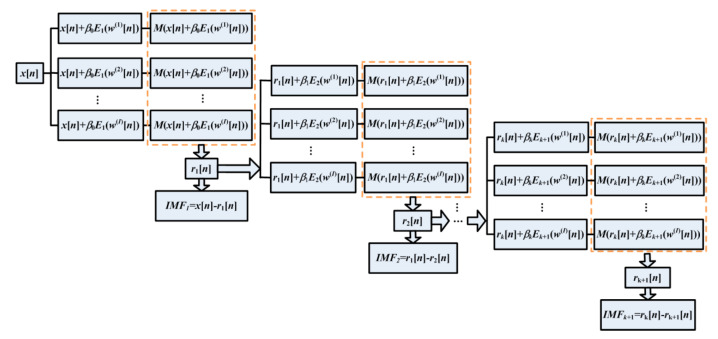
Flow chart of the improved complete ensemble empirical mode decomposition with adaptive noise (ICEEMDAN) [17].

The ICEEMDAN method proposed by Colominas in 2014 is the improvement on the ensemble EMD and complete ensemble EMD [24]. The flow chart of the ICEEMDAN algorithm is described and shown in Figure 1 [5].

In Figure 1, $E_{k}(\cdot )$ demonstrates the generation of the *k*th IMF by EMD, M(⋅) is used for the calculation of local mean, and $w^{\left(i\right)}(i\;=\;1,2,\cdots I)$ is the white Gaussian noise with zero mean unit variance.

### 2.2. ICEEMDAN Energy Entropy

The vibration signal of the rolling bearing can be expressed as the sum of the *n* IMFs and the residual after the ICEEMDAN decomposition, that is:

(1) $$x(t)=\sum_{j=1}^{n}{\mathrm{IMF}}_{j}(t)+r(t)$$

The amplitude energy *E*_1_, *E*_2_, …, *E*_n_ of the IMFs is calculated respectively, that is:

(2) $$E_{j}=\sum_{k=1}^{N}{\left|{\mathrm{IMF}}_{j}(k)\right|}^{2}$$

where *N* is the number of sampling points of the *j*th IMF. Assuming that the energy carried by *r* (*t*) can be ignored, the total energy of the original signal is expressed as:

(3) $$E=\sum_{j=1}^{n}E_{j}=\sum_{j=1}^{n}\sum_{k=1}^{N}|{\mathrm{IMF}}_{j}(k){|}^{2}$$

In order to make the data in the same magnitude, the amplitude energy of each IMF is normalized to facilitate subsequent calculations and reduce the impact of singular data, that is:

(4) $$p_{i}=E_{i}/E$$

Finally, the corresponding ICEEMDAN energy entropy is obtained:

(5) $$H_{EN}=-\sum_{i=1}^{n}p_{i}\mathrm{lg}p_{i}$$

According to this method, the ICEEMDAN energy entropy of the vibration signals under the condition of normal bearing, ball fault, inner and outer race fault are calculated respectively. The results are shown in Table 1.

From Table 1 it can be seen that the energy entropy value obtained after ICEEMDAN decomposition is different when the bearing is in different states. This is because when the bearing has different faults, the frequencies of the corresponding vibration signals and the amplitude energy of the components on different frequency scales change significantly. Therefore, the feature vector constructed by the effective IMF energy entropy after the signal ICEEMDAN decomposition can accurately reflect the corresponding fault information in essence.

## 3. SVM Fault-Diagnosis Model Based on AFSA Optimization

**Figure 2:**
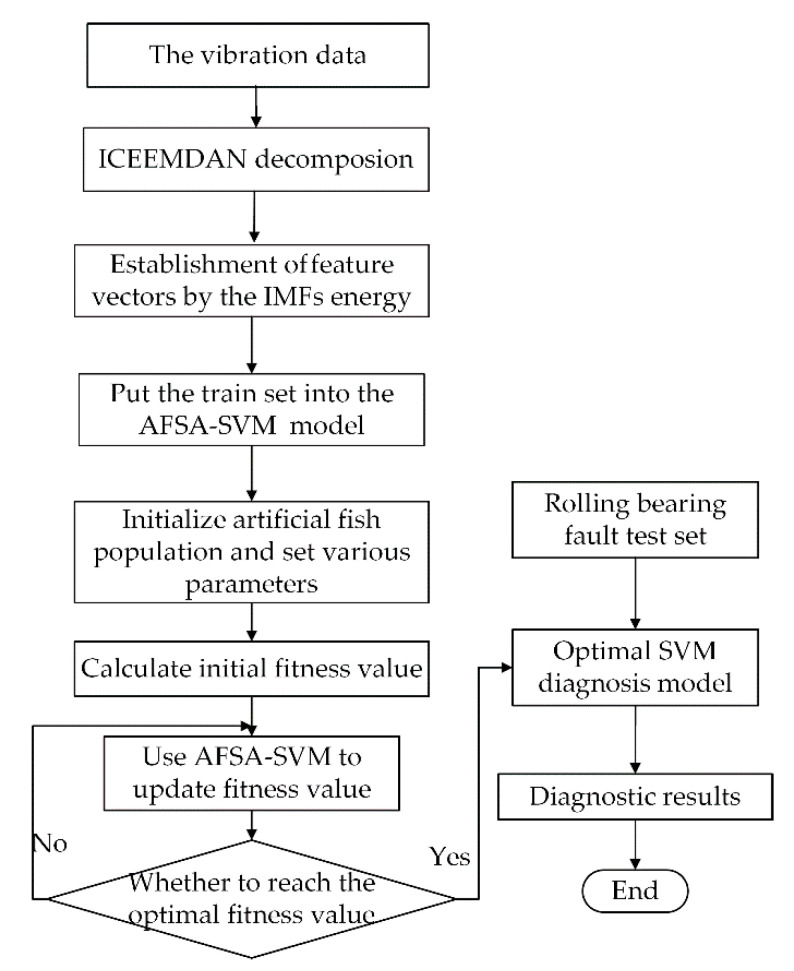
Block diagram of the diagnosis process.

When establishing the SVM diagnostic model with the radial basis function (RBF) as the kernel function, the values of the penalty factor *γ* and the radial basis kernel parameter *σ*^2^ need to be determined. Different parameter values have a great impact on the diagnosis results. Therefore, the artificial fish swarm algorithm (AFSA) [25] is used to iteratively optimize the two parameters to obtain the optimal SVM model. The specific process is shown in Figure 2.

The specific steps are as follows:

**Step 1**  Collect vibration data of various faults of bearings, extract fault characteristics of the data, construct train sets and test sets to test the generalized performance;
**Step 2**  Initialize the fish swarm. Since there are two adjustable parameters, each fish is composed of two dimensions, and the parameters of the algorithm are set;
**Step 3**  Initially set the value range of the penalty factor *γ* and the radial basis kernel parameter *σ*^2^ to be optimized in the SVM;
**Step 4**  After the parameters are initialized, the train samples are used to iteratively optimize the parameters (*γ* and *σ*^2^). Take the mean square error (MSE) of the known fault categories and train results as the fitness function. The optimal SVM model is obtained when the fitness value is the smallest.
**Step 5**  Finally, the diagnosis result is obtained when the test set is input into the optimized SVM diagnostic model.

## 4. Experiment

In order to verify the effectiveness and reliability of the fault-diagnosis method based on ICEEMDAN and AFSA-SVM in the fault diagnosis of the hoist sheave bearing, an experimental study is carried out in this paper.

**Figure 3:**
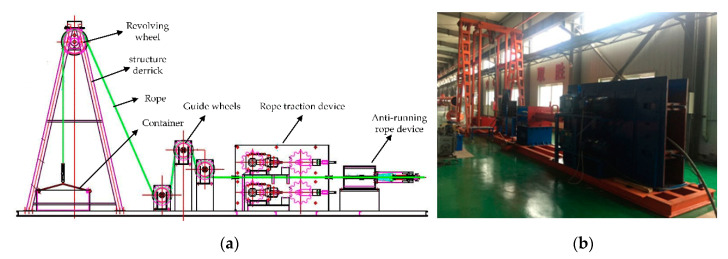
Experimental equipment: (**a**) schematic diagram; (**b**) photo of the equipment.

Since the coal mine multi-rope friction winder is a large-scale revolving equipment and has strict safety assurance requirements, it is hard to carry out the field tests on the coal mine hoist bearings. It is imperative to build a hoist testing setup (as shown in Figure 3) to simulate the working states of the coal mine hoist bearings more truly. Its working principles and load characteristics are similar to the real coal mine hoist. The hoist testing setup was composed of a steel structure derrick with the height of 10 m simulating the derrick of the actual multi-rope friction winder, a revolving wheel device equivalent to the hoist head sheaves, guide wheels and wire rope traction drive device which can make the vertical movement of steel wire rope placed on the friction wheel changed into horizontal motion to control the lifting of the container. The test setup provides driving force through a three-phase asynchronous motor, and the frequency converter is used to control the forward and reverse operation of the wire rope and its operating speed.

**Figure 4:**
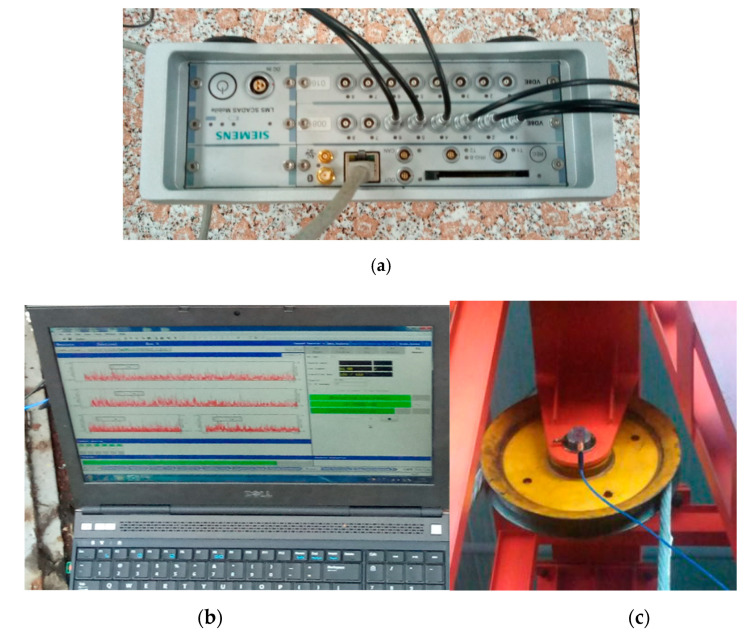
Data acquisition equipment: (**a**) LMSdata acquisition system; (**b**) signal acquisition software; (**c**) installation of sensor.

The bearing conditions of the revolving wheel in this test setup are similar to those of the actual head sheave bearing in multi-rope friction winder. So, the vibration signals were tested under four states through this testing system: the normal condition, the inner race defect, outer race defect, and ball defect of the bearing. The vibration data of the bearing was collected by a three-way accelerometer (PCB356A26, PCB Piezotronics, lnc. Buffalo, New York, NY, USA) with the frequency response range of 1–5 kHz, and transmitted to the LMS data acquisition system (LMS SCADAS Mobile) controlled by the signal acquisition software (LMS Test. Lab, LMS company, Belgium, Brussel), as shown in Figure 4a,b. The three-way accelerometer was mounted on the housing of the bearing cover, as shown in Figure 4c. The sampling frequency is 1000 Hz. The vibration data of twelve seconds are extracted for analysis.

**Figure 5:**
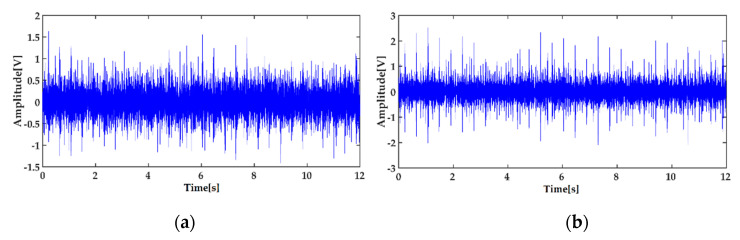
Vibration signal of the inner race fault: (**a**) the collected vibration signal; (**b**) its de-noised signal by MED.

**Figure 6:**
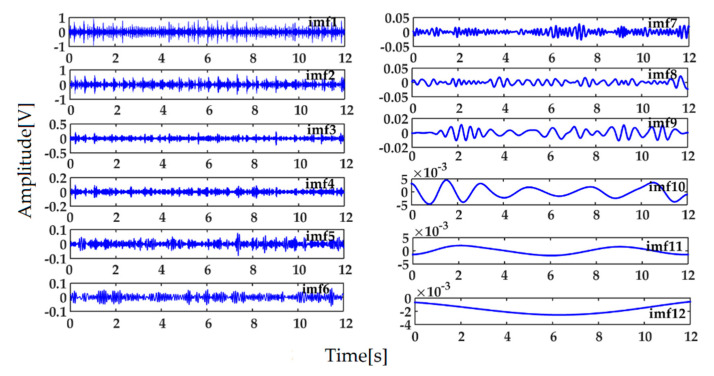
ICEEMDAN decomposition result of the de-noised signal.

In order to reduce the influence of noise on the recognition result, the minimum entropy deconvolution (MED) method is performed on the collected signal to reduce the noise first. The original signal of the inner race fault and its de-noised signal by MED are shown in Figure 5a,b respectively. The ICEEMDAN decomposition result of the de-noised signal of the inner race defect is shown in Figure 6. The calculated energy of each IMF is shown in Table 2.

**Figure 7:**
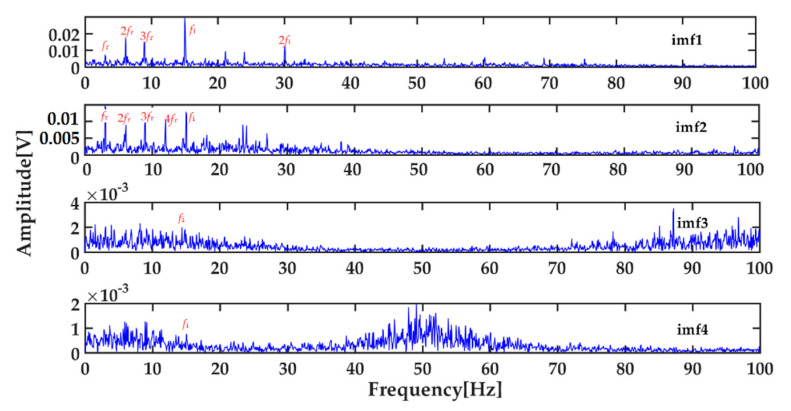
Envelope spectra of the first four IMFs.

As can be seen from Table 2, the first four IMFs contain the main energy (97.9% of total energy) after the de-noised signal is decomposed by ICEEMDAN, and the envelope spectra of them are shown in Figure 7. It can be seen that the envelope spectra of first four IMFs (especially the first two IMFs) contain the main fault features of the bearing inner race from Figure 7. So the feature vector of SVM constructed by the energy entropy of the first four IMFs can essentially reflect the bearing’s corresponding fault.

For the vibration signals of normal bearing, inner race defect, outer race defect, and ball defect, the energy entropy of each IMF after ICEEMDAN decomposition is calculated respectively, and the energy entropy of the first four IMFs is used as the feature vector according to the above method. About 60 sets of feature vectors of each state were extracted, and a total of 240 sets of data were used as train samples. In addition, 20 sets of feature vectors of each state are extracted, and a total of 80 sets of data are used for testing. Because of the large amount of data, Table 3 only lists part of the train sample data used for training.

## 5. Fault-Diagnosis Results

### 5.1. Fault Diagnosis Result without Optimization

**Figure 8:**
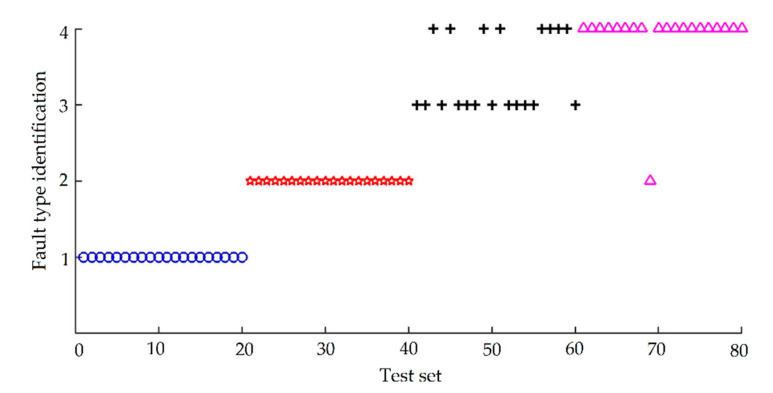
Diagnostic results before support vector machine (SVM) optimization.

In order to highlight the effect of AFSA optimization on improving the classification accuracy of the SVM diagnostic model, the 240 sets of bearing feature vectors are input into the SVM model without optimization. The radial basis function is used as the kernel function in the SVM model without optimization. The penalty factor *γ* and the radial basis kernel parameter *σ*^2^ according to experience are set as 100 and 0.2. After training, 80 groups of data for testing the generalization performance were input into the trained model, and the classification results obtained are shown in Figure 8. Category 1 represents normal bearing, category 2 represents bearing inner race defect, category 3 represents bearing outer race defect, and category 4 represents bearing ball defect. The test results are represented by blue circles, red pentagrams, black crosses, and rose red triangles respectively. The diagnosis results are shown in Table 4.

As can be seen from Table 4, when the two parameters in the support vector machine are not optimized, the diagnostic accuracy of the established model is 87.5%, the mean square error is 0.1625, and the running time is 0.323 s, which shows that the SVM has strong adaptive ability in pattern recognition and is very suitable for intelligent fault diagnosis, but the accuracy of diagnosis needs to be further improved.

### 5.2. Fault-Diagnosis Result of AFSA-SVM

**Figure 9:**
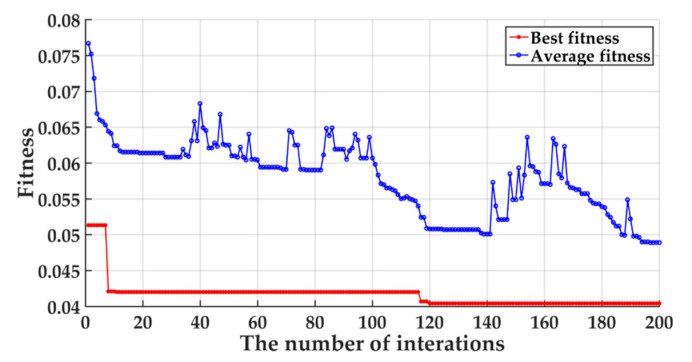
Fitness curve of artificial fish swarm algorithm (AFSA)-SVM algorithm.

The parameters of the AFSA optimization algorithm are set as follows: the maximum number of iterations is 50, the size of the population is 10, the maximum number of foraging trials is 10, the congestion factor *δ* is 0.618, the artificial fish perception distance is 0.5, the moving step is 0.1, and the search dimension is 2. The initial values of the penalty factor *γ* and the radial basis kernel parameter *σ*^2^ are both set to 0.1, and their value range is set to [0.1, 1000]. The average fitness and optimal fitness of the algorithm diagnosis results are shown in Figure 9 respectively.

It can be seen from Figure 9 that the average fitness and the best fitness gradually decrease with the increase of the number of iteration steps. When the iteration step is 200, the average fitness decreased from 0.0767 to 0.0489, with a decrease of 36.3%. At the same time, the best fitness value decreased from 0.0513 to 0.0404, with a decrease of 21.3%. It can be seen from the iteration trend that the average fitness value is constantly approaching the best fitness value, which shows that the SVM diagnosis model is continuously optimized. After the program is completed, the optimal SVM diagnosis model is obtained, with the optimal parameters *γ* = 5.0702 and *σ*^2^ = 10.5966.

**Figure 10:**
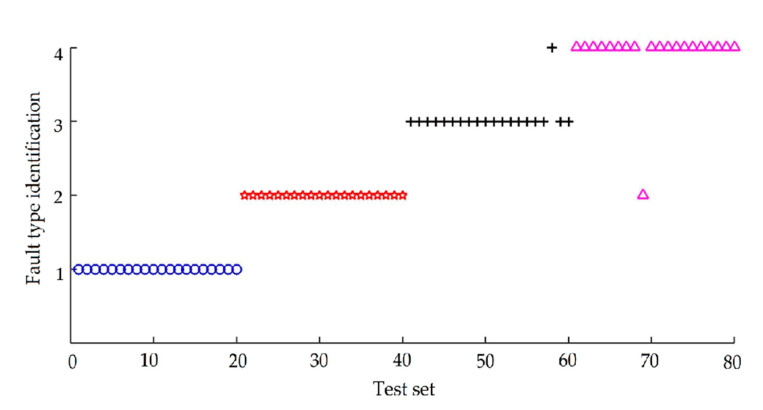
Diagnostic results of AFSA-SVM.

The optimal classification accuracy of SVM diagnosis model is obtained by using AFSA to optimize the two parameters penalty factor *γ* and radial basis kernel parameter *σ*^2^ of the SVM model. Total of 240 sets of bearing feature vectors are input into the optimized AFSA-SVM model. About 80 sets of bearing feature vectors are used to test the generalization performance as the data to be tested. The classification result is shown in Figure 10, and the meaning of category label is the same as in Figure 8.

It can be seen from Figure 10 that the accuracy of SVM model after AFSA optimization has been greatly improved. Only two samples of 80 samples are judged wrong, and the rest have been correctly output, with the diagnostic accuracy of 97.5%, which shows that the optimized SVM diagnosis model can accurately and effectively diagnose the fault category of bearing.

The diagnostic results of the SVM model without optimization and the optimized SVM model are compared, as shown in Table 5.

It can be seen from Table 5 that the SVM model after the artificial fish swarm algorithm optimization has improved greatly in terms of accuracy and mean square error performance. Although the running time is slightly larger than the model before optimization, the fault-diagnosis accuracy rate was increased to 97.5%.

In order to verify the generalization ability of the AFSA-SVM algorithm, different numbers of train samples and test set were constructed. Table 6 shows the results of the different train and test data.

It can be seen from Table 6 that when the number of test data is equal, the accuracy of the AFSA-SVM diagnostic model increases with the number of train samples; when the train samples are equal, the increase in the test set can further improve the diagnostic accuracy, which shows that the generalization ability of the algorithm is relatively strong. Under the condition that the number of train samples is not large, the algorithm achieves a high diagnostic accuracy rate of 99.1%, which indicates that the algorithm has advantages in a small sample condition.

**Figure 11:**
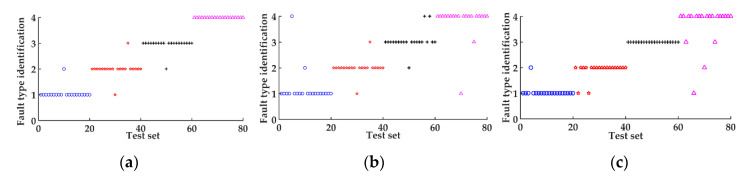
Diagnostic results: (**a**) The result of particle swarm optimization-least squares support vector machine (PSO-LSSVM) combined with ICEEMDAN energy entropy; (**b**) the result of empirical mode decomposition (EMD) energy entropy with AFSA-SVM; (**c**) the result of ensemble empirical mode decomposition (EEMD) energy entropy with AFSA-SVM.

In order to verify the effectiveness of the proposed method, the least squares support vector machine (LSSVM) optimized by particle swarm optimization (PSO), combined with feature vectors constructed by ICEEMDAN energy entropy, was used to diagnose the same bearing faults. Besides, the feature vectors constructed by EMD energy entropy and EEMD energy entropy respectively, combined with AFSA-SVM model, are used to verify the effectiveness of ICEEMDAN energy entropy. The diagnostic process is the same as above. The classification results are shown in Figure 11, and the meaning of category label is the same as in Figure 8. The specific diagnostic results for the three feature vector construction methods with the AFSA-SVM model are shown in Table 7.

From Figure 11, it can be seen that the accuracy of the PSO-LSSVM model combined with the feature vectors constructed by ICEEMDAN energy entropy is 95%, inferior to the proposed method in this paper. The accuracy for the feature vectors constructed by EMD energy entropy and EEMD energy entropy with the AFSA-SVM model is 88.75% and 91.25% respectively, both lower than the accuracy of ICEEMDAN energy entropy with AFSA-SVM as can be seen in Table 7. The effectiveness of the proposed method is verified by the comparisons.

## 6. Summary and Conclusions

An intelligent fault-diagnosis method for the hoist sheave bearing is presented in this paper. Because of the fact that the coal mine multi-rope friction winder is a large-scale revolving equipment and has strict safety assurance requirements, it is hard to carry out the field tests on the coal mine hoist bearings. The experiments were carried out on the hoist testing setup whose working principles and load characteristics are similar to the real coal mine hoist. Four states of the bearing were analyzed: the normal condition, the inner race defect, outer race defect, and ball defect of the bearing.

The feature vectors were constructed by the energy entropy of the IMFs derived from ICEEMDAN decomposition based on the fact that different bearing faults have different ICEEMDAN energy entropy.

The SVM was used to diagnose bearings’ faults. In order to improve the diagnosis accuracy, the two parameters penalty factor *γ* and radial basis kernel parameter *σ*^2^ of the SVM were optimized by the artificial fish swarm algorithm in this paper. After optimization, the accuracy of the fault-diagnosis model can reach 97.5%, increasing by 10% compared with the accuracy without AFSA optimization.

This paper provides a method for the intelligent fault diagnosis of large-scale revolving machinery with low speed and heavy load. The disadvantage of this scheme is that the vibration data cannot be collected in some difficult-to-access places (the acoustic analysis is available for this case), while the advantage is that the incipient fault can be detected from the vibration data which are more sensitive to faults.

In the future analysis, the accuracy of the intelligent fault-diagnosis model can be improved by more optimization algorithm and new feature-extraction methods.

## Figures and Tables

**Table 1:** ICEEMDAN energy entropy corresponding to different bearings faults.

Bearing Condition	Normal Bearing	Outer Race Fault	Inner Race Fault	Ball Fault
Energy Entropy	2.0511	0.4431	0.8033	1.2406

**Table 2:** Energy of each intrinsic mode function (IMF).

IMF	IMF1	IMF2	IMF3	IMF4	IMF5	IMF6	IMF7	IMF8
Energy (*E_i_*/*E*)	0.6512	0.2569	0.0620	0.0087	0.0026	0.0015	0.0008	0.0006

**Table 3:** Train sample.

Bearing State	Feature Vector				Fault Type Identification
Normal bearing	0.2619	0.2451	0.1781	0.0219	1
	0.2641	0.2535	0.1739	0.0733	
	0.2594 …	0.2464 …	0.2049 …	0.0779 …	
	0.2593	0.2423	0.1882	0.0352	
	0.2567	0.2428	0.1969	0.0455	
Inner race defect	0.3659	0.2513	0.1589	0.0140	2
	0.3628	0.2418	0.1411	0.0159	
	0.3603 …	0.2426 …	0.1507 …	0.0365 …	
	0.3665	0.2507	0.1394	0.0365	
	0.3653	0.2459	0.1629	0.0412	
Outer race defect	0.3668	0.2374	0.2029	0.0979	3
	0.3678	0.2522	0.2479	0.1145	
	0.3569 …	0.2489 …	0.2639 …	0.1112 …	
	0.3610	0.2436	0.2389	0.1268	
	0.3675	0.2658	0.2507	0.1069	
Ball defect	0.2401	0.2549	0.2309	0.1185	4
	0.2545	0.2672	0.2606	0.1136	
	0.2017 …	0.2967 …	0.2245 …	0.1042 …	
	0.2386	0.2429	0.2354	0.0961	
	0.2247	0.2722	0.2274	0.0919	

**Table 4:** Diagnostic results of SVM without optimization.

Prediction Algorithm	Sample Number (Training + Prediction)	Accuracy for Training Data	Accuracy for Forecast Data	MSE	Running Time
SVM without optimization	240 + 80	100%	87.5%	0.1625	0.323054 s

**Table 5:** Comparison of results before and after optimization.

Prediction Algorithm	Sample Number (Training + Prediction)	Accuracy for Training Data	Accuracy for Forecast Data	MSE	Running Time
SVM	240 + 80	92.5%	87.5%	0.1625	0.32305 s
AFSA-SVM	240 + 80	100%	97.5%	0.041584	4.22307 s

**Table 6:** Comparison of different samples.

Prediction Algorithm	Sample Number (Training + Prediction)	Accuracy for Training Data	Accuracy for Forecast Data	MSE	Running Time
AFSA-SVM	240 + 80	100%	97.5%	0.041584	4.22307 s
	240 + 160	100%	98.2%	0.036632	5.45123 s
	480 + 80	100%	99.1%	0.025306	6.82108 s

**Table 7:** Diagnostic results of different feature vector construction methods.

Feature Vector Construction Methods	Sample Number (Training + Prediction)	Accuracy (%)				
		Normal Bearing	Inner Race Defect	Outer Race Defect	Ball Defect	Overall Accuracy
EMD	240 + 80	90	90	85	90	88.75
energy entropy						
EEMD	240 + 80	95	90	100	80	91.25
energy entropy						
ICEEMDAN energy entropy	240 + 80	100	100	95	95	97.5
